# Nonlinear Correlation Between Prognostic Nutritional Index and Disease Activity of Rheumatoid Arthritis: A Cross‐Sectional Study

**DOI:** 10.1002/iid3.70516

**Published:** 2026-10-06

**Authors:** Lina Leng, Quanyi Tang, Ying Li, Jinfeng Zhang, Yaorong Han, Xiaoli Li

**Affiliations:** ^1^ Department of Rheumatology Xingtai People's Hospital Xingtai China; ^2^ Department of Internal Medicine Graduate School of Hebei Medical University Shijiazhuang China; ^3^ Department of Oncology 82 Group Hospital of Chinese People's Liberation Army Baoding China

**Keywords:** cross‐sectional study, disease activity level, inflammation, nutritional status, prognostic nutritional index, rheumatoid arthritis

## Abstract

**Background:**

The prognostic nutritional index (PNI) is a composite marker reflecting the nutritional and inflammatory status. Its association with disease activity in rheumatoid arthritis (RA) remains incompletely understood. This study aimed to investigate the relationship between PNI and RA disease activity.

**Methods:**

A total of 984 RA patients were enrolled and stratified into three groups according to PNI tertiles. Univariate analysis and linear regression models were performed to evaluate the association between PNI and disease activity scores (DAS28‐ESR/DAS28‐CRP). A two stage (segmented) linear regression model was used to identify potential inflection points in the relationship.

**Results:**

Univariate analysis showed that PNI was significantly negatively correlated with both DAS28‐ESR (β = −0.043, *p* < 0.001) and DAS28‐CRP (β = −0.045, *p* < 0.001). After adjusting for demographic characteristics, inflammatory markers, and therapeutic drugs, this negative correlation remained significant in the fully adjusted model (DAS28‐ESR: β = −0.034, *p* < 0.001; DAS28‐CRP: β = −0.044, *p* < 0.001). Segmented regression identified a non‐linear relationship with an inflection point at approximately 45.0. Below this threshold, the negative association was markedly stronger (DAS28‐ESR: β = −0.073; DAS28‐CRP: β = −0.069). Above the inflection point, the association weakened and became non‐significant for DAS28‐ESR (β = −0.012, *p* > 0.05), while for DAS28‐CRP it remained significant but attenuated (β = −0.020, *p* < 0.05).

**Conclusion:**

PNI is independently negatively associated with RA disease activity, with a notably stronger effect when PNI is below 45. PNI may serve as a simple and effective biomarker for assessing inflammation and nutritional status in RA patients, and may help identify patients with greater nutritional‐inflammatory burden.

## 1 Introduction

Rheumatoid arthritis (RA) is a chronic autoimmune disease characterized by persistent, erosive polyarthritis, affecting approximately 1% of the global population [1, 2]. The global burden of RA has steadily increased, with projections estimating a rise from 1.07 million cases in 2019 to 1.5 million by 2040 [3]. Common manifestations include morning stiffness, fatigue, pain, swelling, and joint damage [4], and RA is also associated with extra‐articular complications such as depression, interstitial lung disease, cardiovascular disease, lymphoma, and hematological abnormalities [5, 6, 7, 8].

The pathogenesis of RA is complex and multifactorial, involving interactions among genetic, hormonal, immunological, and environmental factors [9]. Among these, nutritional factors have garnered increasing attention due to their potential reversibility. Immune dysregulation in RA involves activation of various immune cells that drive joint erosion, synovial angiogenesis, and tissue remodeling [10]. Importantly, malnutrition can exacerbate immune dysfunction and promote inflammatory responses [11]. RA patients generally exhibit poorer nutritional status compared to healthy individuals [12], and malnutrition has been closely linked to increased all‐cause mortality in this population [13]. Thus, both immune and nutritional status should be considered in the diagnosis and prognosis of RA.

Established biomarkers such as C‐reactive protein (CRP), erythrocyte sedimentation rate (ESR), rheumatoid factor (RF), and anti‐citrullinated protein antibodies (ACPA) are indispensable for diagnosing RA and monitoring inflammation. However, they have inherent limitations: CRP and ESR primarily reflect acute systemic inflammation, while RF and ACPA indicate autoimmune status but do not capture the metabolic and nutritional consequences of chronic inflammation [14]. The heterogeneity of RA further underscores the need for complementary biomarkers. Currently, no unified diagnostic standard exists for RA; diagnosis relies mainly on joint symptoms and serological markers [9]. Approximately one‐third of patients are seronegative for traditional autoantibodies such as RF and ACPA [15], and the insidious onset of symptoms [16] can delay diagnosis. Given the interplay between inflammation and nutrition, composite scores derived from routine laboratory parameters—such as the Prognostic Nutritional Index (PNI), which integrates serum albumin and lymphocyte counts—may capture a unique dimension of disease burden.

PNI is calculated as serum albumin (g/L) + 5 × lymphocyte count (× 10^9^/L). Albumin reflects nutritional status, while lymphocyte count reflects immune status. Originally developed to assess nutritional risk and postoperative complications in surgical patients [17], PNI has since been validated as a prognostic marker in various malignancies [18, 19, 20, 21, 22]. Beyond oncology, PNI has demonstrated prognostic value in chronic inflammatory conditions. For instance, it independently predicts disease activity in inflammatory bowel disease [23] and is associated with adverse outcomes and mortality in cardiovascular disease [24]. In autoimmune diseases, an l‐shaped association has been reported between PNI and both RA presence and all‐cause mortality [25]. However, the relationship between PNI and RA disease activity remains incompletely characterized.

This cross‐sectional study aimed to comprehensively investigate the association between PNI and disease activity in RA patients, and to evaluate whether PNI could serve as a simple, cost‐effective complementary marker for comprehensive clinical assessment.

## 2 Methods

### 2.1 Research Design and Population

This cross‐sectional study retrospectively reviewed the medical records of 984 patients with RA who attended Xingtai People's Hospital and met the inclusion criteria. A detailed description of the cohort assembly and data collection timeline has been provided in our previous publication examining the leukocyte‐to‐albumin ratio (LAR) in RA [26]. In brief, the inclusion criteria were: (1) age ≥ 18 years; and (2) fulfillment of the 2010 American College of Rheumatology/European League Against Rheumatism (ACR/EULAR) classification criteria for RA [27]. The exclusion criteria were: age < 18 years (*n* = 6); pregnant or lactating women; concomitant other autoimmune diseases (*n* = 76); history of malignant tumors or hematological disorders (*n* = 18); severe liver or kidney dysfunction (*n* = 34); acute or chronic infections (*n* = 42); and missing clinical or laboratory data (*n* = 39). The patient selection process is illustrated in Figure 1.

**Figure 1:**
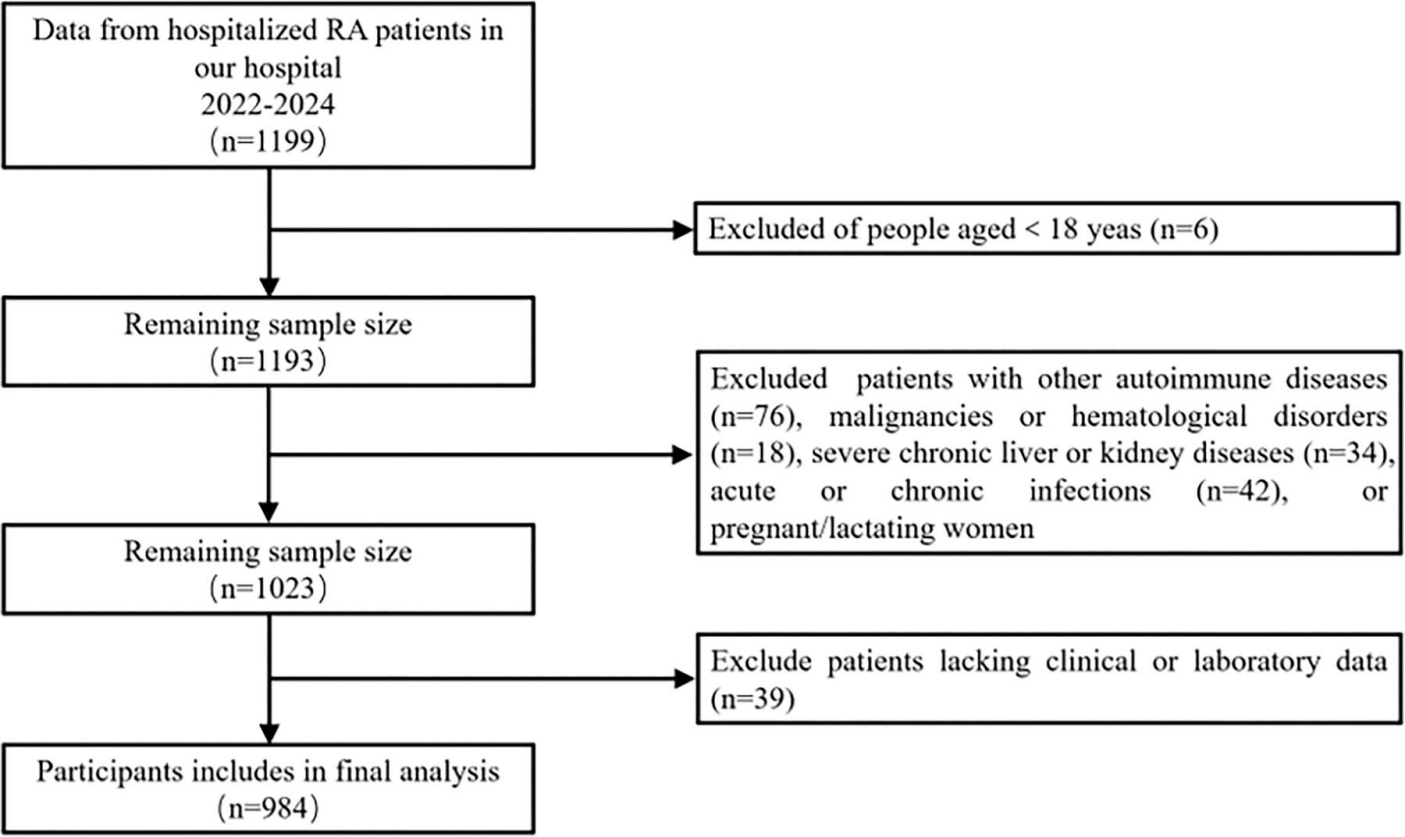
Flowchart of participant selection.

The study was approved by the Ethics Committee of Xingtai People's Hospital (approval number: 2025[031]) and conducted in accordance with the principles of the Declaration of Helsinki. Written informed consent was obtained from all participants.

### 2.2 Data Collection

The data collection protocol and variable definitions used in this study were consistent with those described in our prior work investigating inflammatory‐nutritional composite markers in this cohort [26]. Briefly, demographic and clinical variables included sex, age, body mass index (BMI), and disease duration (DD). Treatment‐related variables included glucocorticoid use, disease‐modifying antirheumatic drugs (DMARDs) and their types (0, 1, or ≥ 2), and biologic DMARD use. Laboratory parameters included ESR, CRP, white blood cell count (WBC), neutrophil count (Neut), lymphocyte count (Lymph), monocyte count (Mono), red blood cell count (RBC), hemoglobin (HGB), red blood cell distribution width (RDW), platelet count (PLT), platelet distribution width (PDW), albumin, and globulin. PNI was calculated as: PNI = serum albumin (g/L) + 5 × lymphocyte count (×10^9^/L). Blood samples were collected on the same day as DAS28 assessment. All tests were performed in the certified hospital laboratory following standard procedures. Disease activity assessment: Calculate 28 joint disease activity scores (DAS28‐ESR/DAS28‐CRP). DAS28‐ESR and DAS28‐CRP scores were retrospectively extracted from electronic medical records, where they had been documented by attending rheumatologists during routine clinical care following standardized joint examination procedures based on EULAR/ACR guidelines. To ensure data quality, only patients with complete DAS28 documentation were included, and extracted data were verified by two independent researchers.

### 2.3 Statistical Analysis

All statistical analyses were performed using IBM SPSS Statistics (version 25.0) and R software (version 4.3.3). Nonlinear relationship analysis was conducted using the ‘segmented’ package (version 2.0‐0) in R, and figures were generated using the ‘ggplot2’ package. A two‐tailed *p* < 0.05 was considered statistically significant.

Descriptive analysis: Patients were divided into tertiles (T1, T2, T3) based on their PNI values. Continuous variables were tested for normality using the Shapiro–Wilk test. Normally distributed variables were presented as mean ± standard deviation and compared across groups using one‐way analysis of variance (ANOVA). Non‐normally distributed variables were presented as median (interquartile range) and compared using the Kruskal–Wallis H test. Categorical variables were expressed as frequencies (percentages) and compared using the chi‐square test.

Univariate analysis: Simple linear regression was used to examine the association between each potential predictor (demographic characteristics, laboratory markers, treatment‐related factors, and PNI) and disease activity (DAS28‐ESR and DAS28‐CRP). Results were presented as unstandardized β coefficients with 95% confidence intervals (CIs).

Multivariate analysis: Three hierarchical linear regression models were constructed to evaluate the independent association between PNI and disease activity: Crude model: Unadjusted. Model I: Adjusted for sex, age, disease duration (DD), and body mass index (BMI). Model II: Further adjusted for neutrophil count, monocyte count, red blood cell count, hemoglobin, platelet count, platelet distribution width, globulin, glucocorticoid use, biologic agent use, and number of DMARD types. PNI was analyzed both as a continuous variable and as tertiles (with the lowest tertile as reference). Multicollinearity was assessed using variance inflation factors (VIF), with VIF < 5 considered acceptable. In the fully adjusted model (Model II), a total of 13 covariates were included. According to the events‐per‐variable (EPV) rule of thumb requiring 10–15 cases per covariate, the minimum required sample size was estimated to be 195 cases. Our final cohort of 984 patients provided more than adequate statistical power.

Nonlinear relationship analysis: A two‐stage (segmented) linear regression model was used to explore potential nonlinear relationships between PNI and DAS28 scores. The inflection point was determined by maximizing the likelihood, and its significance was evaluated using the log‐likelihood ratio test. Segmented regression coefficients with 95% CIs were reported for PNI values below and above the inflection point.

Subgroup analysis: Subgroup analyses were performed stratified by disease phase, defined based on DAS28‐ESR scores: remission (< 2.6) and active disease (≥ 2.6). Due to the limited sample size in the remission group (*n* = 27), only descriptive statistics were presented for this subgroup. For the active disease group, multivariate linear regression was repeated using the same covariates as in Model II.

Sensitivity analysis: To assess the robustness of our findings, sensitivity analyses were performed by excluding potentially correlated variables—specifically neutrophil count, monocyte count, and globulin—individually and in combination. The resulting β coefficients for PNI were compared with those from the full model to evaluate the stability of the estimates.

Study design consideration: It should be noted that due to the cross‐sectional design of this study, all analyses presented here examine associations rather than causal relationships. The regression models identify factors associated with disease activity but cannot establish temporal sequences or causality.

## 3 Results

### 3.1 Baseline Characteristics

This study included 984 RA patients, including 769 females (78.20%) with a median age of 59 years. According to the third quartile of PNI, all patients were divided into three groups: T1 (low PNI group, *n* = 327), T2 (medium PNI group, *n* = 328), and T3 (high PNI group, *n* = 329). As shown in Table 1, there were significant differences (*p* < 0.05) among the three groups of patients in multiple baseline features. Compared with the T3 group, patients in the T1 group (low PNI) were older, had a lower proportion of females, had higher levels of inflammatory markers (ESR, CRP, WBC), and significantly lower levels of nutrition related markers (lymphocytes, red blood cells, hemoglobin, albumin). More importantly, the disease activity levels (DAS28‐ESR and DAS28‐CRP) of T1 group were significantly higher than those of T2 and T3 groups (*p* < 0.001).

**Table 1:** General feature description according to tertiles of PNI.

Characteristics	Total	Tertiles of PNI			
		T1(25.95–42.29)	T2(42.29–47.70)	T3(47.70–64.99)	*p* value
*N*	984	327	328	329	
Sex, %					0.024
Female	769 (78.20%)	239 (73.09%)	266 (81.10%)	264 (80.24%)	
Male	215 (21.80%)	88 (26.91%)	62 (18.90%)	65 (19.76%)	
Age (year)	59.00 (50.00, 68.00)	61.00 (53.00, 69.00)	58.00 (47.00, 67.00)	57.00 (49.00, 65.50)	< 0.001
DD (month)	60.00 (12.00, 120.00)	60.00 (12.00, 132.00)	61.50 (19.25, 120.00)	60.00 (12.00, 120.00)	0.654
BMI (kg/m^2^)	23.67 (21.48, 26.04)	23.28 ± 3.38	23.77 (21.48, 26.14)	24.36 ± 3.36	< 0.001
Smoking, %					0.024
No	929 (94.4%)	301 (92.05%)	318 (96.95%)	310 (94.22%)	
Yes	55 (5.6%)	26 (7.95%)	10 (3.05%)	19 (5.78%)	
Alcohol use, %					0.220
No	966 (98.2%)	321 (98.17%)	325 (99.09%)	320 (97.26%)	
Yes	18 (1.8%)	6 (1.83%)	3 (0.91%)	9 (2.74%)	
Glucocorticoids, %					0.145
No	880 (89.40%)	284 (86.85%)	295 (89.94%)	301 (91.49%)	
Yes	104 (10.60%)	43 (13.15%)	33 (10.06%)	28 (8.51%)	
DMARDs, %					0.017
No	513 (52.10%)	180 (55.05%)	150 (45.73%)	183 (55.62%)	
Yes	471 (47.90%)	147 (44.95%)	178 (54.27%)	146 (44.38%)	
Type of DMARDs, %					0.021
0	513 (52.10%)	180 (55.05%)	150 (45.73%)	183 (55.62%)	
1	352 (35.80%)	108 (33.03%)	127 (38.72%)	117 (35.56%)	
≥ 2	119 (12.10%)	39 (11.92%)	51 (15.55%)	29 (8.82%)	
BDMARDs, %					0.598
No	922 (93.70%)	310 (94.80%)	306 (93.29%)	306 (93.01%)	
Yes	62 (6.30%)	17 (5.20%)	22 (6.71%)	23 (6.99%)	
ESR (mm/H)	57.00 (34.00, 87.00)	78.00 (51.00, 109.00)	54.00 (30.00, 83.75)	52.00 (27.00, 68.50)	< 0.001
CRP (mg/L)	27.85 (7.29, 48.77)	43.12 (21.69, 72.15)	25.64 (7.06, 43.33)	11.39 (3.49, 33.98)	< 0.001
WBC (*10^9^/L)	6.15 (4.97, 7.76)	6.00 (4.61, 7.53)	5.82 (4.78, 7.30)	6.76 (5.52, 8.22)	< 0.001
Neut (*10^9^/L)	3.97 (2.87, 5.27)	4.06 (2.84, 5.39)	3.69 (2.76, 4.95)	4.10 (3.00, 5.36)	0.024
Lymph (*10^9^/L)	1.54 (1.20, 1.93)	1.21 (0.97, 1.52)	1.48 (1.25, 1.81)	1.96 (1.61, 2.41)	< 0.001
Mono (*10^9^/L)	0.44 (0.34, 0.58)	0.45 (0.34, 0.59)	0.44 (0.34, 0.56)	0.44 (0.33, 0.58)	0.920
RBC (*10^12^/L)	3.87 ± 0.50	3.63 ± 0.48	3.87 ± 0.45	4.12 ± 0.43	< 0.001
HGB (g/L)	111.00 (100.00, 123.00)	101.33 ± 16.32	111.02 ± 14.72	119.90 ± 15.69	< 0.001
RDW (fL)	43.90 (41.10, 47.00)	44.50 (41.60, 47.90)	44.15 (41.40, 47.20)	43.10 (40.45, 45.95)	< 0.001
PLT (*10^9^/L)	278.00 (225.00, 346.00)	284.00 (225.00, 359.00)	274.00 (216.00, 342.00)	278.00 (233.00, 344.00)	0.124
PDW (fL)	10.40 (9.30, 11.60)	10.00 (8.90, 11.10)	10.50 (9.50, 11.70)	10.60 (9.70, 12.20)	< 0.001
Albumin (g/L)	36.87 ± 4.91	32.60 (29.90, 34.40)	37.20 ± 2.42	41.41 ± 3.13	< 0.001
Globulin (g/L)	32.10 (28.50, 36.00)	32.10 (28.60, 36.80)	31.45 (28.35, 35.88)	32.15 ± 4.91	0.596
DAS28‐ESR	4.51 (3.91, 5.07)	4.88 (4.34, 5.30)	4.32 ± 0.85	4.31 (3.61, 4.75)	< 0.001
DAS28‐CRP	3.79 (3.20, 4.32)	4.22 (3.62, 4.58)	3.63 ± 0.79	3.53 (2.89, 4.05)	< 0.001

Abbreviations: BDMARDs, biologics; BMI, body mass index; CRP, high‐sensitivity C‐reactive protein; DD, disease duration; DMARDs, disease‐modifying antirheumatic drugs; ESR, erythrocyte sedimentation rate; HGB, hemoglobin; Lymph, lymphocyte count; Mono, monocyte count; Neut, neutrophil count; PLT, platelet; PDW, platelet distribution width; PNI, prognostic nutritional index; RBC, red blood cell; RDW, red blood cell distribution width; WBC, white blood cell count.

### 3.2 Univariate Analysis of Factors Associated with Disease Activity

The results of univariate linear regression analysis (Table 2) showed that PNI was significantly negatively correlated with DAS28‐ESR (β = −0.043, 95% CI: −0.051 to −0.035, *p* < 0.001) and DAS28‐CRP (β = −0.045, 95% CI: −0.053 to −0.037, *p* < 0.001). In addition, age, inflammatory markers (ESR, CRP, WBC, Neut, Mono, PLT), and globulin were positively correlated with higher DAS28 scores; And males, use of DMARDs/biologics, higher hemoglobin, albumin, and PDW are associated with lower DAS28 scores.

**Table 2:** The results of univariate analysis.

Characteristics	Statistics	DAS28‐ESR β (95% CI)	*p*_ERS_value	DAS28‐CRP β (95% CI)	*p*_CRP_ value
Sex, *N* (%)					
Female	769 (78.20%)	Ref		Ref	
Male	215 (21.80%)	−0.133 (−0.258, 0.008)	0.037	−0.309 (−0.429, −0.189)	< 0.001
Age (year)	59.00 (50.00, 68.00)	0.013 (0.009, 0.018)	< 0.001	0.012 (0.008, 0.016)	< 0.001
DD (month)	60.00 (12.00, 120.00)	0.000 (0.000, 0.001)	0.428	0.000 (0.000, 0.001)	0.310
BMI (kg/m^2^)	23.67 (21.48, 26.04)	−0.008 (−0.023, 0.007)	0.310	−0.004 (−0.019, 0.010)	0.546
Smoking, %					
No	929 (94.4%)	Ref		Ref	
Yes	55 (5.6%)	0.161 (−0.064, 0.387)	0.161	0.315 (0.097, 0.533)	0.005
Alcohol use, %					
No	966 (98.2%)	Ref		Ref	
Yes	18 (1.8%)	−0.034 (−0.421, 0.354)	0.864	0.128 (−0.247, 0.504)	0.503
Glucocorticoids, *N* (%)					
No	880 (89.40%)	Ref		Ref	
Yes	104 (10.60%)	−0.082 (−0.251, 0.087)	0.339	−0.063 (−0.227, 0.101)	0.450
DMARDs, *N* (%)					
No	513 (52.10%)	Ref		Ref	
Yes	471 (47.90%)	−0.310(−0.412, −0.208)	< 0.001	−0.327(−0.426, −0.228)	< 0.001
Type of DMARDs					
0	513 (52.10%)	Ref		Ref	
1	352 (35.80%)	−0.489(−0.651, −0.327)	< 0.001	−0.434 (−0.592, −0.277)	< 0.001
≥ 2	119 (12.10%)	−0.249(−0.360, −0.139)	< 0.001	−0.291 (−0.398, −0.184)	< 0.001
BDMARDs					
No	922 (93.70%)	Ref		Ref	
Yes	62 (6.30%)	−0.366(−0.579, −0.154)	0.001	−0.334 (−0.540, −0.128)	0.002
ESR (mm/H)	57.00 (34.00, 87.00)	0.019 (0.018, 0.020)	< 0.001	0.015 (0.014, 0.016)	< 0.001
CRP (mg/L)	27.85 (7.29, 48.77)	0.010 (0.009, 0.011)	< 0.001	0.013 (0.012, 0.014)	< 0.001
WBC (*10^9^/L)	6.15 (4.97, 7.76)	0.095 (0.073, 0.118)	< 0.001	0.124 (0.103, 0.145)	< 0.001
Neut (*10^9^/L)	3.97 (2.87, 5.27)	0.125 (0.099, 0.150)	< 0.001	0.162 (0.138, 0.186)	< 0.001
Lymph (*10^9^/L)	1.54 (1.20, 1.93)	−0.028 (−0.115, 0.060)	0.536	−0.037 (−0.122, 0.048)	0.395
Mono (*10^9^/L)	0.44 (0.34, 0.58)	0.496 (0.223, 0.770)	< 0.001	0.773 (0.510, 1.036)	< 0.001
RBC (*10^12^/L)	3.87 ± 0.50	−0.381(−0.482, −0.279)	< 0.001	−0.210 (−0.310, −0.110)	< 0.001
HGB (g/L)	111.00 (100.00, 123.00)	−0.013(−0.016, −0.010)	< 0.001	−0.009 (−0.012, −0.006)	< 0.001
RDW (fL)	43.90 (41.10, 47.00)	0.001 (−0.005, 0.007)	0.742	0.003 (−0.002, 0.009)	0.218
PLT (*10^9^/L)	278.00 (225.00, 346.00)	0.003 (0.002, 0.003)	< 0.001	0.003 (0.002, 0.003)	< 0.001
PDW (fL)	10.40 (9.30, 11.60)	−0.091(−0.116, −0.065)	< 0.001	−0.073(−0.098, −0.048)	< 0.001
Albumin (g/L)	36.87 ± 4.91	−0.066(−0.075, −0.056)	< 0.001	−0.069(−0.078, −0.059)	< 0.001
Globulin (g/L)	32.10 (28.50, 36.00)	0.078 (0.007, 0.086)	< 0.001	0.063 (0.055, 0.071)	< 0.001
PNI	44.97 ± 6.18	−0.043 (−0.051, −0.035)	< 0.001	−0.045 (−0.053, −0.037)	< 0.001

Abbreviations: BDMARDs, biologics; BMI, body mass index; CRP, high‐sensitivity C‐reactive protein; DD, disease duration; DMARDs, disease‐modifying antirheumatic drugs; ESR, erythrocyte sedimentation rate; HGB, hemoglobin; Lymph, lymphocyte count; Mono, monocyte count; Neut, neutrophil count; RBC, red blood cell; RDW, red blood cell distribution width; PDW, platelet distribution width; PLT, platelet; PNI, prognostic nutritional index; WBC, white blood cell count.

### 3.3 Multivariate Linear Regression Analysis of PNI and Disease Activity

In the multivariate adjusted model (Table 3), the negative correlation between PNI and DAS28 scores remains robust. After fully adjusting for all confounding factors in Model II, for every unit increase in PNI, the DAS28‐ESR score significantly decreased by 0.034 points (β = −0.034, 95% CI: −0.043 to −0.024, *p* < 0.001), and the DAS28‐CRP score significantly decreased by 0.044 points (β = −0.044, 95% CI: −0.053 to −0.035, *p* < 0.001). Taking T1 group as a reference, the DAS28 scores of T2 and T3 groups were significantly reduced (all *p* < 0.001), and there was a significant dose‐response trend (*P* for trend< 0.001). Collinearity diagnostics revealed no serious multicollinearity, with all VIF values below 5 (e.g., neutrophil count VIF = 1.549, monocyte count VIF = 1.375, globulin VIF = 1.264). Sensitivity analyses excluding these correlated variables individually or in combination yielded consistent results, with PNI β coefficients changing by less than 0.003 and remaining statistically significant (all *p* < 0.001) (Table 4).

**Table 3:** Multivariate linear regression results of association between PNI and DAS28.

Exposure	Crude model β (95% CI)	*p* value	Adjust I β (95% CI)	*p* value	Adjust II β (95% CI)	*p* value
DAS28‐ESR						
PNI	−0.043 (−0.051, −0.035)	< 0.001	−0.040 (−0.048, −0.032)	< 0.001	−0.023 (−0.031, −0.016)	< 0.001
PNI (Tertiles)						
T1	Ref		Ref		Ref	
T2	−0.450 (−0.571, −0.328)	< 0.001	−0.408 (−0.530, −0.286)	< 0.001	−0.266 (−0.369, −0.163)	< 0.001
T3	−0.567 (−0.689, −0.446)	< 0.001	−0.524 (−0.646, −0.402)	< 0.001	−0.354 (−0.479, −0.229)	< 0.001
*P* for trend		< 0.001		< 0.001		< 0.001
DAS28‐CRP						
PNI	−0.045 (−0.053, −0.037)	< 0.001	−0.042 (−0.050, −0.034)	< 0.001	−0.035 (−0.042, −0.027)	< 0.001
PNI (Tertiles)						
T1	Ref		Ref		Ref	
T2	−0.437 (−0.554, −0.320)	< 0.001	−0.393 (−0.510, −0.276)	< 0.001	−0.286 (−0.390, −0.182)	< 0.001
T3	−0.605 (−0.722, −0.488)	< 0.001	−0.563 (−0.680, −0.446)	< 0.001	−0.480 (−0.606, −0.354)	< 0.001
*P* for trend		< 0.001		< 0.001		< 0.001

*Note:* Crude model model adjust for. Adjust I model adjust for: sex; age; disease duration; BMI. Adjust II model adjust for: sex; age; BMI; neutrophil count; monocyte count; red blood cell; hemoglobin; platelet; platelet distribution width; globulin; glucocorticoids; biologics; type of DMARDs. ^†^All variance inflation factors (VIF) in the final models were below 5, indicating no serious multicollinearity.

Abbreviation: PNI, prognostic nutritional index.

**Table 4:** Sensitivity analysis excluding potentially correlated variables.

Model	PNI β for DAS28‐ESR (95% CI)	*p* value	PNI β for DAS28‐CRP (95% CI)	*p* value
Full Model II (with all covariates)	−0.023 (−0.031, −0.016)	< 0.001	−0.035 (−0.043, −0.027)	< 0.001
Excluding neutrophil count	−0.024 (−0.032, −0.016)	< 0.001	−0.036 (−0.044, −0.028)	< 0.001
Excluding monocyte count	−0.023 (−0.031, −0.016)	< 0.001	−0.035 (−0.042, −0.027)	< 0.001
Excluding globulin	−0.025 (−0.034, −0.017)	< 0.001	−0.036 (−0.044, −0.028)	< 0.001
Excluding neutrophil count, monocyte count and globulin	−0.026 (−0.035, −0.017)	< 0.001	−0.037 (−0.045, −0.029)	< 0.001

*Note:* Model II were adjusted for sex; age; BMI; neutrophil count; monocyte count; red blood cell; hemoglobin; platelet; platelet distribution width; globulin; glucocorticoids; biologics; type of DMARDs.

### 3.4 Nonlinear Relationship Analysis

We further explored the relationship between PNI and DAS28 scores using a two‐stage linear regression model (Table 5, Figure 2 and Figure 3). After adjusting for confounding factors, we found a significant non‐linear relationship between PNI and DAS28 scores (likelihood ratio test *p* < 0.001). For DAS28‐ESR: When PNI < 45.016, for every additional unit of PNI, the DAS28‐ESR score significantly decreased by 0.073 points (β = −0.073, 95% CI: −0.091 to −0.055). When PNI > 45.016, the strength of this negative correlation significantly weakens, and the increase in PNI no longer significantly reduces the DAS28‐ESR score (β = −0.012, 95% CI: −0.031 to 0.007, *p* > 0.05). For DAS28‐CRP: When PNI < 45.015, for every additional unit of PNI, the DAS28‐CRP score significantly decreased by 0.069 points (β = −0.069, 95% CI: −0.086 to −0.052). When PNI > 45.015, although the association is still significant, the effect value decreases significantly (β = −0.020, 95% CI: −0.038 to −0.002).

**Table 5:** The result of two‐piecewise linear regression model of PNI with DAS28‐ESR and DAS28‐CRP.

	DAS28‐ESR β (95% CI)	DAS28‐CRP β (95% CI)
Fitting model by standard linear regression	4.426 (4.377, 4.474)	3.716 (3.670, 3.763)
Fitting model by two‐piecewise linear regression		
Inflection points of PNI	45.016	45.015
< Inflection point	−0.073 (−0.091, −0.055)	−0.069 (−0.086, −0.052)
> Inflection point	−0.012 (−0.031, 0.007)	−0.020 (−0.038, −0.002)
*p* for log likelihood ratio test	< 0.001	< 0.001

*Note:* The models were adjusted for sex; age; BMI; neutrophil count; monocyte count; red blood cell; hemoglobin; platelet; platelet distribution width; globulin; glucocorticoids; biologics; type of DMARDs.

Abbreviation: PNI, prognostic nutritional index.

**Figure 2:**
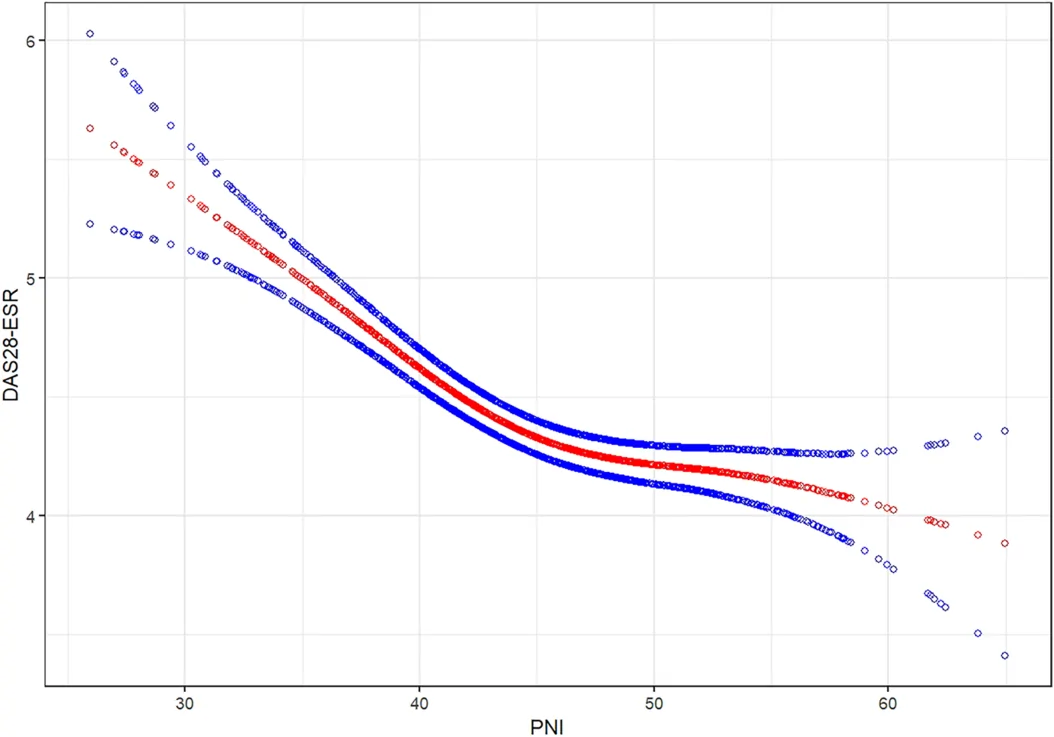
A nonlinear relationship of PNI with DAS28‐ESR. The model was adjusted for sex; age; BMI; neutrophil count; monocyte count; red blood cell; hemoglobin; platelet; platelet distribution width; globulin; glucocorticoids; biologics; type of DMARDs. The solid line represents the fitted piecewise regression line, and the dashed lines represent the 95% confidence intervals. The inflection point was estimated at 45.016. The Davies test confirmed significant nonlinearity (*p* < 0.001).

**Figure 3:**
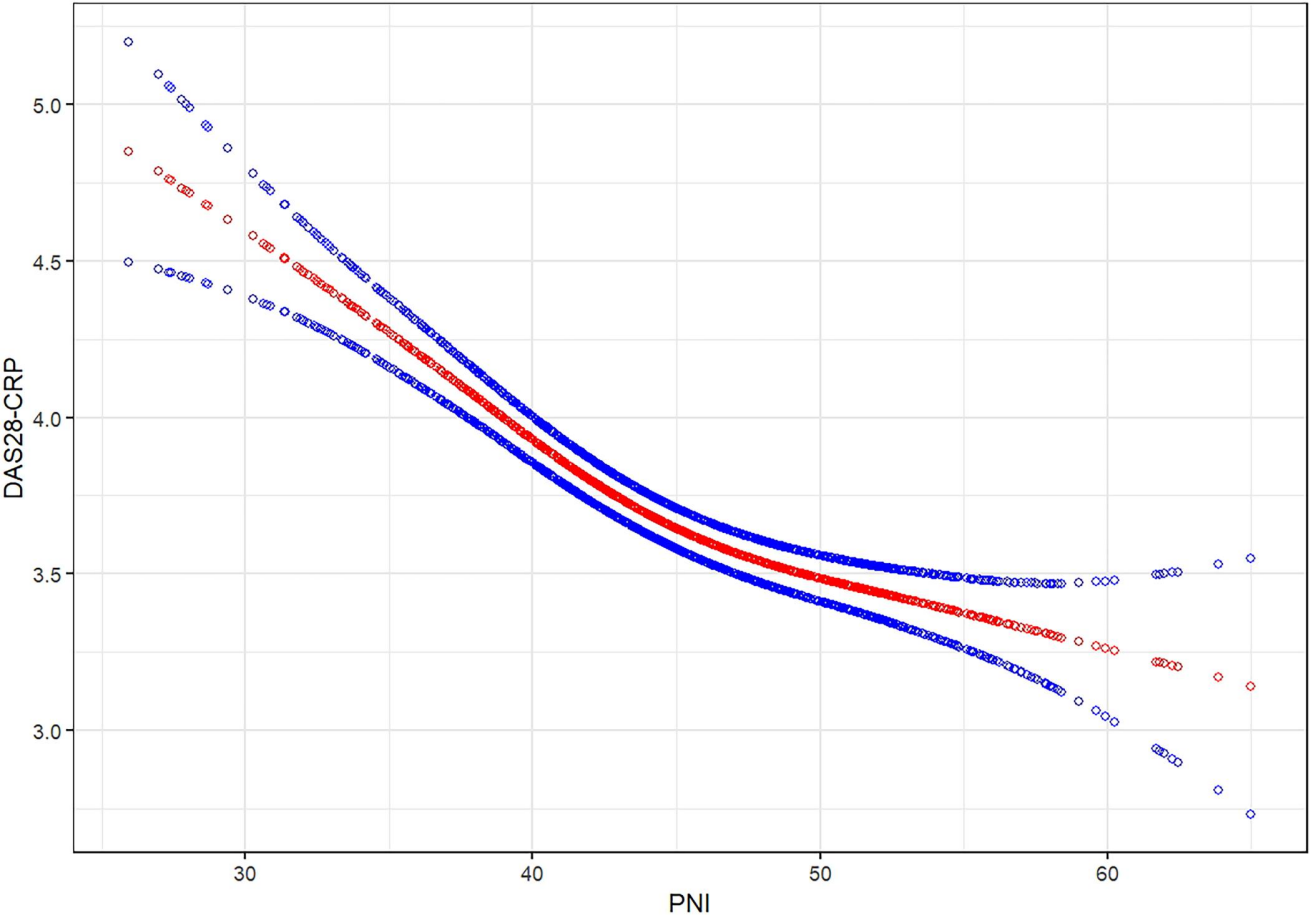
A nonlinear relationship of PNI with DAS28‐CRP. Note: The model was adjusted for sex; age; BMI; neutrophil count; monocyte count; red blood cell; hemoglobin; platelet; platelet distribution width; globulin; glucocorticoids; biologics; type of DMARDs. The solid line represents the fitted piecewise regression line, and the dashed lines represent the 95% confidence intervals. The inflection point was estimated at 45.015. The Davies test confirmed significant nonlinearity (*p* < 0.001).

### 3.5 Subgroup Analysis by Disease Phase

Subgroup analysis stratified by disease phase showed that in the active disease group (*n* = 957), PNI remained significantly associated with DAS28‐ESR and DAS28‐CRP, consistent with the main analysis. In the remission group (*n* = 27), only descriptive statistics were performed due to the small sample size (Table 6).

**Table 6:** Subgroup analysis of the association between PNI and disease activity by disease phase.

Characteristics	Remission group (DAS28‐ESR < 2.6)	Active disease group (DAS28‐ESR ≥ 2.6)
*N*	27	957
PNI	45.95 (43.36, 48.35)^a^	44.91 ± 6.19^b^
DAS28‐ESR	2.40 (2.10, 2.53)^a^	4.54 (3.95, 5.09)^a^
DAS28‐CRP	2.09 ± 0.53^b^	3.83 (3.24, 4.33)^a^
Association between PNI and disease activity^c^		
	Remission group	Active disease group
DAS28‐ESR	Descriptive statistics only^d^	β = −0.023 (−0.030, −0.016)
DAS28‐CRP	Descriptive statistics only^d^	β = −0.034 (−0.041, −0.026)

*Note:* Data are presented as median (Q1, Q3) or mean ± SD.

Abbreviations: DAS28‐CRP, Disease Activity Score in 28 joints ‐ C‐reactive protein; DAS28‐ESR, Disease Activity Score in 28 joints ‐ Erythrocyte Sedimentation Rate; PNI, prognostic nutritional index.

^a^ Variables with non‐normal distribution are presented as median (Q1, Q3).

^b^ Variables with normal distribution are presented as mean ± SD.

^c^ Active disease group used multivariate linear regression models adjusted for sex; age; BMI; neutrophil count; monocyte count; red blood cell; hemoglobin; platelet; platelet distribution width; globulin; glucocorticoids; biologics; type of DMARDs.

^d^ Due to the limited sample size in the remission group (*n* = 27), only descriptive statistics are presented, and multivariate regression analysis was not performed.

## 4 Discussion

The purpose of this study is to evaluate the association between PNI and RA disease activity, and to find that PNI is significantly independently negatively correlated with DAS28‐ESR and DAS28‐CRP. After adjusting for potential confounding variables, this negative correlation still exists. And there is a clear non‐linear relationship in this association. The present investigation is part of a research program examining the utility of composite inflammatory‐nutritional indices in RA using this cohort. While our previous work in this cohort focused on the leukocyte‐to‐albumin ratio (LAR)—an index that integrates total leukocyte count with albumin—and reported a positive nonlinear association with DAS28 scores [26], the current study addresses a distinct scientific question: the association between PNI (albumin + 5 × lymphocyte count) and RA disease activity. Despite sharing the same patient population and analytical approach, the two studies examine conceptually different exposures and yield distinct clinical insights—LAR captures global inflammatory burden via total leukocytes, whereas PNI specifically integrates the lymphocyte‐mediated adaptive immune component with nutritional reserve. Therefore, the present findings should be interpreted as complementary to our earlier LAR analysis rather than redundant.

The negative association between PNI and RA disease activity can be explained by various underlying pathophysiological mechanisms. First, serum albumin is an important indicator reflecting systemic inflammation and nutritional status, playing roles in antioxidant activity, maintenance of endothelial stability, and regulation of immune and inflammatory responses [28]. Existing evidence suggests that serum albumin levels are significantly lower in RA patients compared to the healthy population [29]. RA is characterized by persistently elevated pro‐inflammatory cytokines, particularly interleukin‐6 (IL‐6) and tumor necrosis factor‐alpha (TNF‐α). These cytokines directly suppress hepatic albumin synthesis by inhibiting albumin gene transcription [30]. Concurrently, chronic inflammation increases systemic protein catabolism and resting energy expenditure, leading to a state of “rheumatoid cachexia” where patients lose muscle mass despite normal or even increased caloric intake [31]. Importantly, hypoalbuminemia itself perpetuates the inflammatory state by impairing antioxidant capacity and reducing the binding and clearance of pro‐inflammatory molecules [32]. Second, lymphocytes are primarily produced by lymphoid organs and play a crucial role in immune responses, serving as key components in the initiation and exacerbation of autoimmune diseases [33]. RA is essentially a progressive systemic autoimmune disease characterized by the breakdown of immune tolerance. Specifically, there is an imbalance between pro‐inflammatory T helper 17 (Th17) cells and anti‐inflammatory regulatory T cells (Tregs), with a relative increase in Th17 cells and decreased Treg function [34]. Chronic inflammation also promotes lymphocyte apoptosis in peripheral lymphoid organs, leading to relative lymphopenia that correlates with disease severity [35]. Furthermore, immunosuppressive therapies commonly used in RA (such as methotrexate and biologic agents) can directly affect lymphocyte counts, making this parameter a useful indicator of both disease activity and treatment response. Therefore, as a composite index, PNI integrates the nutritional and immune dimensions, more accurately capturing the overall pathophysiological status of RA patients and aiding in the identification of high‐risk individuals for personalized management.

Previous studies have investigated the association between PNI and RA disease activity. Kılıç et al. [36] conducted a cross‐sectional study in 116 RA patients (along with 90 healthy controls) and reported significant negative correlations between PNI and DAS28‐ESR using standard linear regression, identifying an optimal PNI cutoff of 40 for discriminating active disease. Similarly, Öz et al. [37] evaluated 138 RA patients and found that PNI was significantly lower in patients with moderate‐to‐high disease activity (DAS28‐ESR > 3.2) compared to those with remission/low activity, with a cutoff value of 43.01 and an odds ratio of 0.85 for predicting disease activity. Both studies provided valuable evidence supporting the utility of PNI as a simple marker for disease activity in RA. However, they were constrained by relatively modest sample sizes (116 and 138, respectively) and relied exclusively on standard linear or logistic regression models, which assume a constant relationship between PNI and disease activity across its entire range. These approaches inherently cannot detect potential threshold effects or nonlinear patterns. In contrast, our study leveraged a substantially larger cohort (*n* = 984) and, for the first time, applied segmented (two‐piecewise) linear regression analysis to uncover a nonlinear relationship between PNI and DAS28 scores, with a consistent inflection point at approximately PNI = 45.0 in both DAS28‐ESR and DAS28‐CRP models. This threshold has direct clinical implications: below 45, each PNI unit increase yields a 0.07‐point DAS28 reduction, which is 3.5 times greater than the effect above the threshold. Notably, for DAS28 CRP both segments were statistically significant, yet the slope decreased from –0.069 below the inflection point to –0.020 above it (a 71% reduction). The Davies test confirmed that this change is significant (*p* < 0.001), indicating a true nonlinear relationship—the effect of PNI does not disappear, but its strength substantially diminishes after the threshold. In addition, our subgroup analysis confirmed that the PNI‐disease activity relationship is robust in the active disease population (97% of patients), but could not be assessed in remission due to limited sample size—a finding that itself highlights the real‐world distribution of disease activity. These contributions provide a stronger evidence base for using PNI as a complementary marker in RA management.

Of course, this study also has some limitations. Firstly, the design of cross‐sectional studies cannot infer the causal relationship between PNI and disease activity. Prospective longitudinal studies and interventional trials are warranted to establish the temporal dynamics of these relationships and to evaluate whether nutritional interventions can modify disease activity. Secondly, as a single‑center study conducted in a Chinese population, our findings may not be directly generalizable to other ethnic or geographical groups. Future multicenter studies involving diverse populations are needed to validate the generalizability of the observed associations. Thirdly, although DAS28 assessments followed standardized protocols, potential inter‐rater variability cannot be completely excluded due to the retrospective nature of data collection. However, the parallel findings for both DAS28‐ESR and DAS28‐CRP support the reliability of our observations. Fourth, the small number of patients in remission (*n* = 27, 2.7%) limited our ability to perform robust multivariate analysis in this subgroup. Although the association between PNI and disease activity was consistent in the active disease population, which constituted the vast majority of our cohort, future studies with larger remission samples are needed to confirm whether this relationship holds in patients with well‐controlled disease. Finally, PNI is a simple composite marker based on albumin and lymphocyte count and does not capture more detailed nutritional parameters such as dietary intake, energy balance, or body composition (e.g., muscle mass). Future studies incorporating these variables (e.g., via food frequency questionnaires, bioelectrical impedance analysis) could provide a more comprehensive understanding of the relationship between nutritional status and RA disease activity.

## 5 Conclusion

In summary, this study confirms that PNI is independently and negatively associated with RA disease activity and reveals for the first time the existence of nonlinear threshold effects between them. PNI, as a simple, economical, and easily applicable composite indicator in clinical practice, can effectively reflect the inflammatory load and nutritional immune status of RA patients, helping doctors quickly identify patients with greater nutritional‐inflammatory burden who may require closer attention to nutritional status. Prospective studies are needed to determine whether interventions targeting nutritional status can influence disease activity.

## Author Contributions

**Lina Leng:** conceptualization, data curation, formal analysis, investigation, methodology, resources, software, supervision, validation, visualization, writing – original draft, writing – review and editing. **Quanyi Tang:** conceptualization, data curation, investigation, methodology, software, visualization, writing – original draft. **Ying Li:** conceptualization, investigation, methodology, visualization, writing – original draft. **Jinfeng Zhang:** conceptualization, investigation, methodology, software, visualization. **Yaorong Han:** conceptualization, investigation, methodology, software, visualization. **Xiaoli Li:** conceptualization, funding acquisition, methodology, project administration, writing – review and editing, resources, visualization.

## Funding

The authors have nothing to report.

## Ethics Statement

This study received approval from the Research Ethics Committee of Xingtai People's Hospital (approval number: 2025[031]). The studies were conducted in accordance with the local legislation and institutional requirements. The participants provided their written informed consent to participate in this study.

## Conflicts of Interest

The authors declare no conflicts of interest.

## Publisher's Note

All claims expressed in this article are solely those of the authors and do not necessarily represent those of their affiliated organizations, or those of the publisher, the editors and the reviewers. Any product that may be evaluated in this article, or claim that may be made by its manufacturer, is not guaranteed or endorsed by the publisher.

## Supporting information


Supporting File 1

Supporting File 2

## Data Availability

The data that support the findings of this study are available on request from the corresponding author. The data are not publicly available due to privacy or ethical restrictions. The original contributions presented in the study are included in the article material, further inquiries can be directed to the corresponding authors.
