# Supplementary material for: Nonlinear Correlation Between Prognostic Nutritional Index and Disease Activity of Rheumatoid Arthritis: A Cross‐Sectional Study
Source: Immun Inflamm Dis. 2026 Oct 6;14(10):e70516. doi: 10.1002/iid3.70516 (PMC13640532; doi:10.1002/iid3.70516)
Supplement: Supplementary file 1 [file IID3-14-e70516-s001.docx]

**Supplementary Table S1. Sensitivity analysis excluding potentially correlated variables.**

| **Model** | **PNI β for DAS28-ESR**  **(95% CI)** | **P value** | **PNI β for DAS28-CRP**  **(95% CI)** | **P value** |
| --- | --- | --- | --- | --- |
| Full Model II (with all covariates) | -0.023 (-0.031, -0.016) | ＜0.001 | -0.035 (-0.043, -0.027) | ＜0.001 |
| Excluding neutrophil count | -0.024 (-0.032, -0.016) | ＜0.001 | -0.036 (-0.044, -0.028) | ＜0.001 |
| Excluding monocyte count | -0.023 (-0.031, -0.016) | ＜0.001 | -0.035 (-0.042, -0.027) | ＜0.001 |
| Excluding globulin | -0.025 (-0.034, -0.017) | ＜0.001 | -0.036 (-0.044, -0.028) | ＜0.001 |
| Excluding neutrophil count, monocyte count and globulin | -0.026 (-0.035, -0.017) | ＜0.001 | -0.037 (-0.045, -0.029) | ＜0.001 |

Note: Model II were adjusted for sex; age; BMI; neutrophil count; monocyte count; red blood cell; hemoglobin; platelet; platelet distribution width; globulin; glucocorticoids; biologics; type of DMARDs.

**Supplementary Table 2.** **Subgroup analysis of the association between PNI and disease activity by disease phase**

| Characteristics | Remission group  (DAS28-ESR < 2.6) | Active disease group  (DAS28-ESR ≥ 2.6) |
| --- | --- | --- |
| N | 27 | 957 |
| PNI | 45.95 (43.36, 48.35) ¹ | 44.91 ± 6.19 ² |
| DAS28-ESR | 2.40 (2.10, 2.53) ¹ | 4.54 (3.95, 5.09) ¹ |
| DAS28-CRP | 2.09 ± 0.53 ² | 3.83 (3.24, 4.33) ¹ |
| **Association between PNI and disease activity³** | | |
|  | Remission group | Active disease group |
| DAS28-ESR | Descriptive statistics only ⁴ | β = -0.023 (-0.030, -0.016) |
| DAS28-CRP | Descriptive statistics only ⁴ | β = -0.034 (-0.041, -0.026) |

**Note:** Data are presented as median (Q1, Q3) or mean ± SD.

¹ Variables with non-normal distribution are presented as median (Q1, Q3).

² Variables with normal distribution are presented as mean ± SD.

³ Active disease group used multivariate linear regression models adjusted for sex; age; BMI; neutrophil count; monocyte count; red blood cell; hemoglobin; platelet; platelet distribution width; globulin; glucocorticoids; biologics; type of DMARDs.

⁴ Due to the limited sample size in the remission group (n=27), only descriptive statistics are presented, and multivariate regression analysis was not performed.

**Abbreviations**: *PNI* prognostic nutritional index, *DAS28-ESR* Disease Activity Score in 28 joints - Erythrocyte Sedimentation Rate*, DAS28-CRP* Disease Activity Score in 28 joints - C-reactive protein.
